# Case Report: Thoracoscopic repair of a residual inferior sinus venosus-type defect with right lower pulmonary vein–inferior vena cava communication: intraoperative support using real-time 3D transesophageal echocardiography

**DOI:** 10.3389/fcvm.2025.1568703

**Published:** 2025-05-13

**Authors:** Tianzong Li, Yiran Luo, Qin Liang, Kui Hu, Chao Wang

**Affiliations:** ^1^ Department of Cardiology, Cardiac Interventional Radiology, Guizhou Provincial People’s Hospital, Guiyang, China; ^2^ Cardiac Function Unit, Department of Cardiology, Guizhou Provincial People’s Hospital, Guiyang, China; ^3^ Department of Cardiac Surgery, Guizhou Provincial People’s Hospital, Guiyang, China; ^4^ Department of Radiology, Guizhou Provincial People’s Hospital, Guiyang, China

**Keywords:** three-dimensional real-time transesophageal echocardiography, thoracoscopic cardiac surgery, multimodality imaging, residual sinus venosus defect, inferior vena cava, right lower pulmonary vein

## Abstract

**Background:**

Residual venous defects after atrial septal defect (ASD) repair may remain asymptomatic for years and can be misinterpreted as partial anomalous pulmonary venous return. Accurate intraoperative imaging is essential to confirm the anatomy and guide repair, particularly in minimally invasive surgery.

**Case presentation:**

A 28-year-old woman with no cardiopulmonary symptoms was referred after an abnormal electrocardiographic findings during a routine physical examination. She had undergone ASD repair at age 3. Transthoracic echocardiography revealed right heart enlargement, severe tricuspid regurgitation, and a suspected communication between right lower pulmonary vein (RLPV) and inferior vena cava (IVC), later confirmed by cardiac CT. She underwent thoracoscopic repair with tricuspid annuloplasty. Real-time 3D transesophageal echocardiography (RT-3D TEE) was used as an intraoperative aid to confirm the location of the residual RLPV–IVC communication, to support the placement of guidewire and catheter, and to assist in post-repair assessment. The surgery was completed successfully with complications, and follow-up imaging showed complete resolution of the defect.

**Conclusion:**

In this case, RT-3D TEE provided effective intraoperative support during thoracoscopic cardiac surgery by enhancing spatial orientation and improving procedural confidence. While not essential in all cases, it may serve as a valuable assistant in anatomically complex or visually limited situations.

## Introduction

Residual or misdiagnosed venous connections after atrial septal defect (ASD) repair may occur and can remain asymptomatic for years. Precise anatomic localization is essential during reoperation, especially in minimally invasive settings where direct visualization is limited. Three-dimensional transesophageal echocardiography (3D TEE) has emerged as a powerful intraoperative tool to assist repair of complex cardiac anomalies.

## Case report

**Figure 1:**
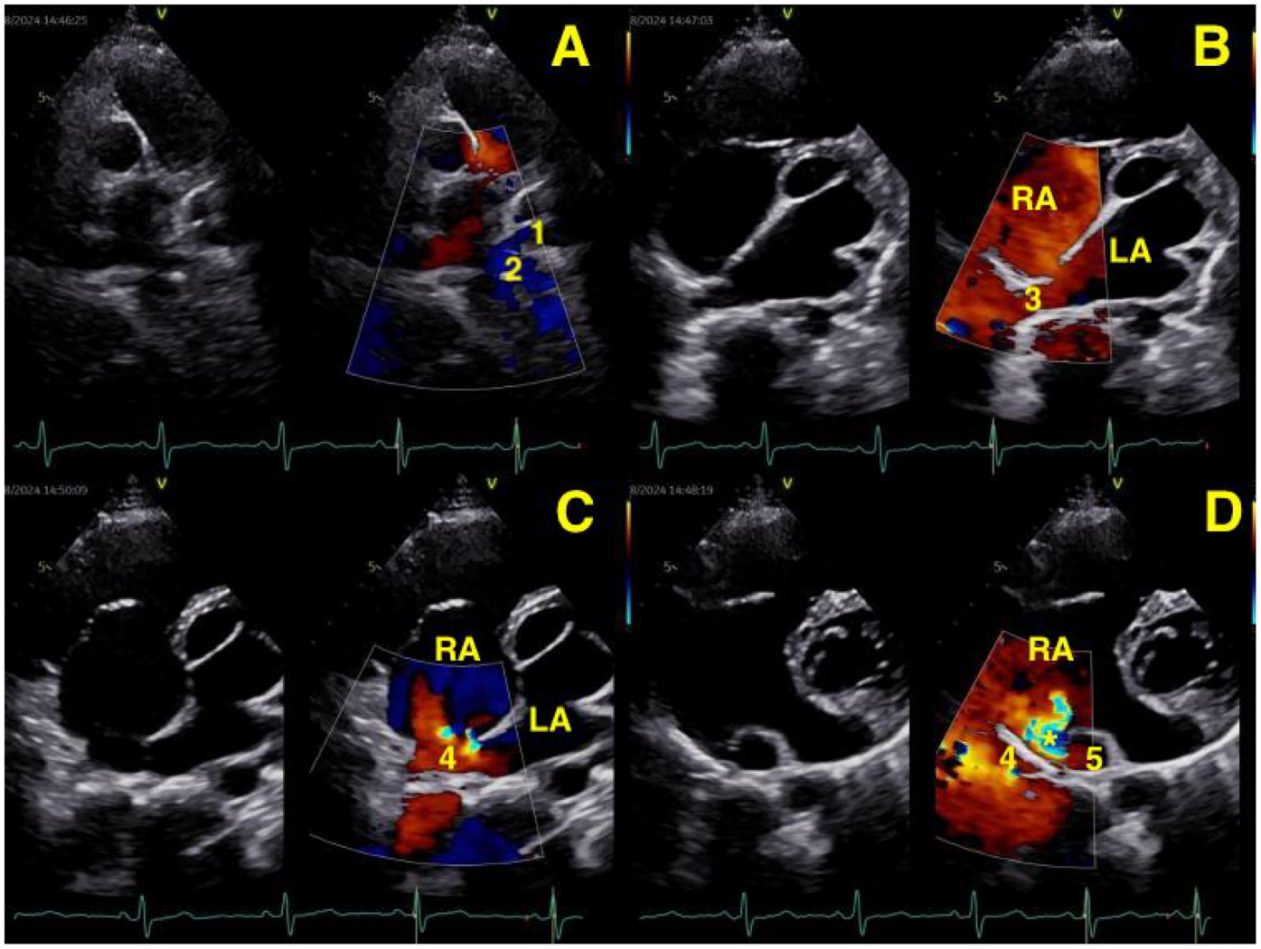
**(A,B)** Transthoracic echocardiography (TTE) showing the left lower pulmonary vein (1), the left upper pulmonary vein (2), and the right upper pulmonary vein (3) entering the left atrium. **(C,D)** TTE showed the right inferior pulmonary vein (4) draining into the left atrium, with Color Doppler revealing a bright, abnormal flow signal at this site. An orifice (*) representing an abnormal communication between the dilated the right lower pulmonary vein (4) and inferior vena cava (5) is visualized below the posterior wall of the right atrium, adjacent to the coronary sinus.

**Figure 2:**
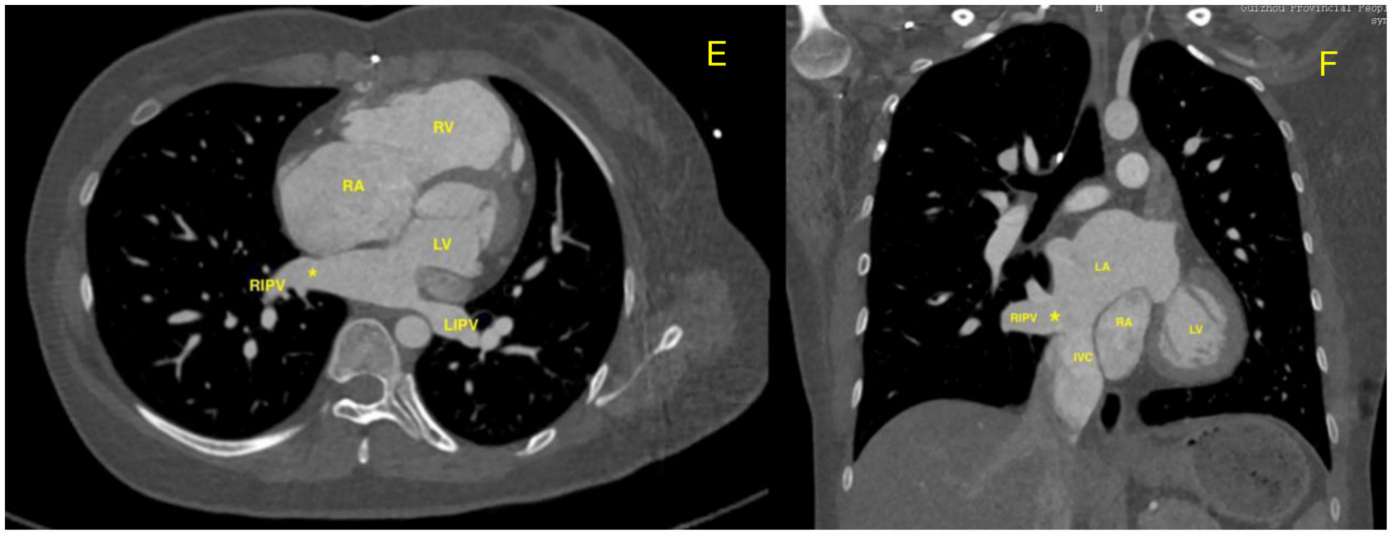
**(E,F)** Contrast-enhanced CT (cross-sectional and coronal views) showing the abnormal communication(*) between the right lower pulmonary vein and the inferior vena cava. The inferior vena cava drains normally into the right atrium; the apparent proximity to the left atrium is due to imaging overlap.

**Figure 3:**
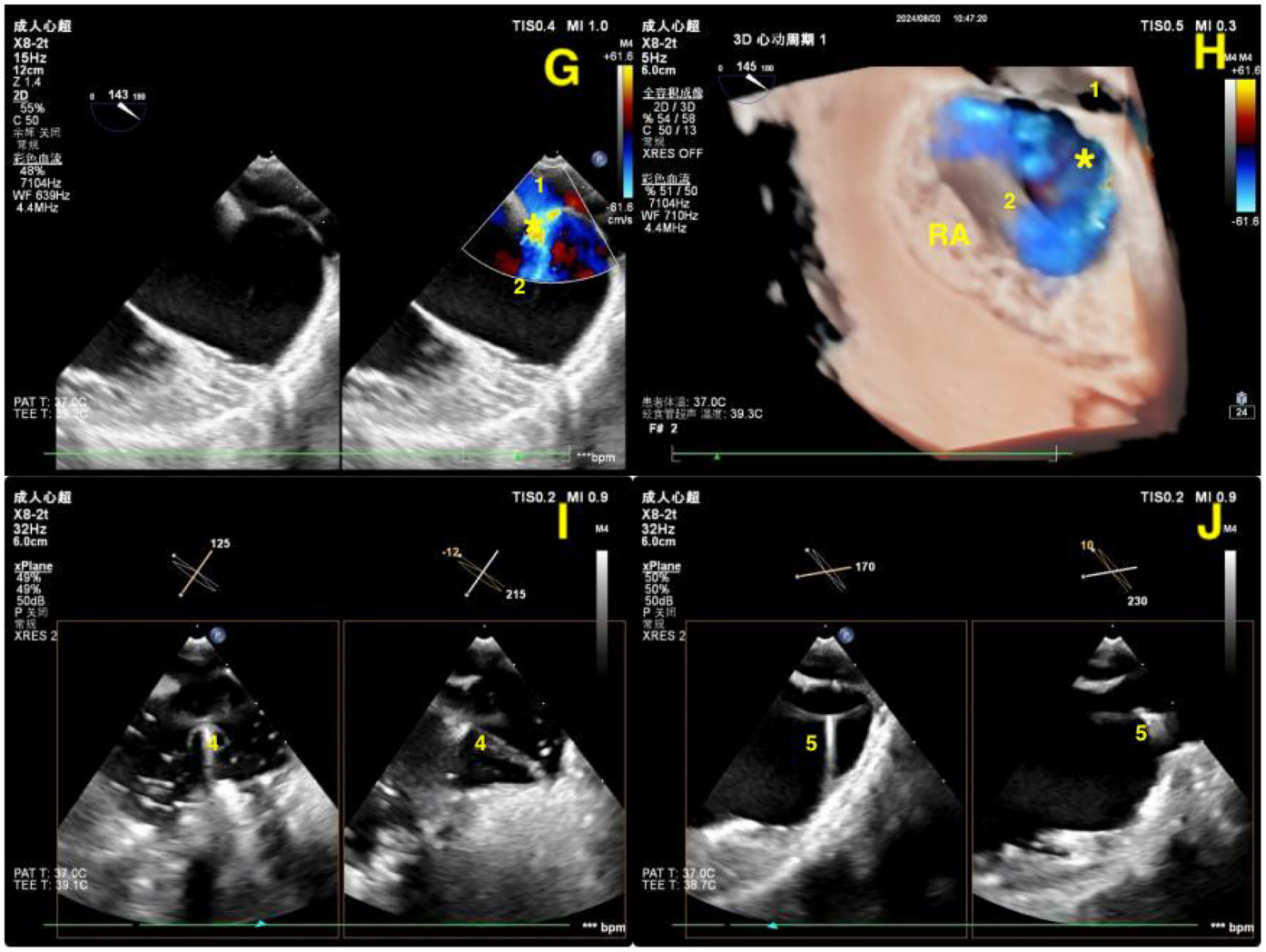
**(G,H)** Intraoperative 2D and real-time 3D transesophageal echocardiography (RT-3D TEE) images illustrating the abnormal connection (*) between the right lower pulmonary vein (1) and the inferior vena cava (2) during thoracoscopic surgery. **(I,J)** X-plane mode 2D TEE images demonstrating real-time positioning of the drainage tube (4) and guidewire (5) along their intended anatomical pathways. RA, right atrium.

A 28-year-old woman with no cardiopulmonary symptoms was referred for further evaluation after abnormal after a routine physical examination at an outside hospital revealed electrocardiographic abnormalities, including right ventricular hypertrophy, incomplete right bundle branch block, and right axis deviation. She had undergone surgical repair of an atrial septal defect (ASD) at the age of 3. Transthoracic echocardiography (TTE) revealed right heart enlargement, severe tricuspid regurgitation, and mild pulmonary arterial hypertension. The atrial septal patch was not clearly visualized. Three pulmonary veins—the left upper, left lower, and right upper—were confirmed to drain normally into the left atrium. However, color Doppler imaging suggested an abnormal venous connection between the right lower pulmonary vein (RLPV) and the inferior vena cava (IVC), raising suspicion of a residual inferior venous communication near the interatrial septum (Figures 1A–D). This diagnosis was further confirmed by computed tomography (CT), which clearly demonstrated an abnormal communication between the right lower pulmonary vein (RLPV) and the inferior vena cava (IVC), with direct wall-to-wall continuity adjacent to the interatrial septum (Figures 2E,F). In addition, the proximal segment of the IVC appeared dilated, causing partial posterior wall distortion of the right atrium and forming a finger-like cavity extending posterosuperiorly. The patient was scheduled for minimally invasive total thoracoscopic repair with tricuspid annuloplasty. Real-time three-dimensional transesophageal echocardiography (RT-3D TEE) was utilized intraoperatively as an adjunct tool to confirm the anatomical relationship prior to right atrial exposure and cardiopulmonary bypass. Color Doppler imaging with RT-3D TEE revealed oxygenated blood from the RLPV draining into the IVC and subsequently into the right atrium through a 7–10 mm residual venous communication. The atrial septum appeared intact, and the morphology and function of both atrioventricular valves were preserved, supporting surgical planning.After thoracoscopic entry and pericardial dissection, cardiopulmonary bypass was established via femoral and superior vena cava cannulation. Direct thoracoscopic visualization was limited by intracardiac blood pooling. Using true-view color mode and X-plane imaging, RT-3D TEE accurately guided intra-atrial orientation, delineated the location of the RLPV–IVC connection, and visualized dynamic positioning of the drainage tube, central venous catheter, and guidewire (Figures 3G–J; Supplementary Videos S1–S4). A right atriotomy was performed parallel to the atrioventricular groove. After establishing cardiopulmonary bypass via femoral and superior vena cava cannulation and evacuating intracardiac blood, an enlarged communication was identified between the right lower pulmonary vein (RLPV) and the inferior vena cava (IVC), located just superior to the IVC orifice. The atrial septum was intact. The residual communication was closed using continuous 4-0 polypropylene sutures. Tricuspid annuloplasty was completed with implantation of a 30 mm Medtronic Contour Ring using interrupted sutures along the annulus. A saline test confirmed minimal residual regurgitation.

**Figure 4:**
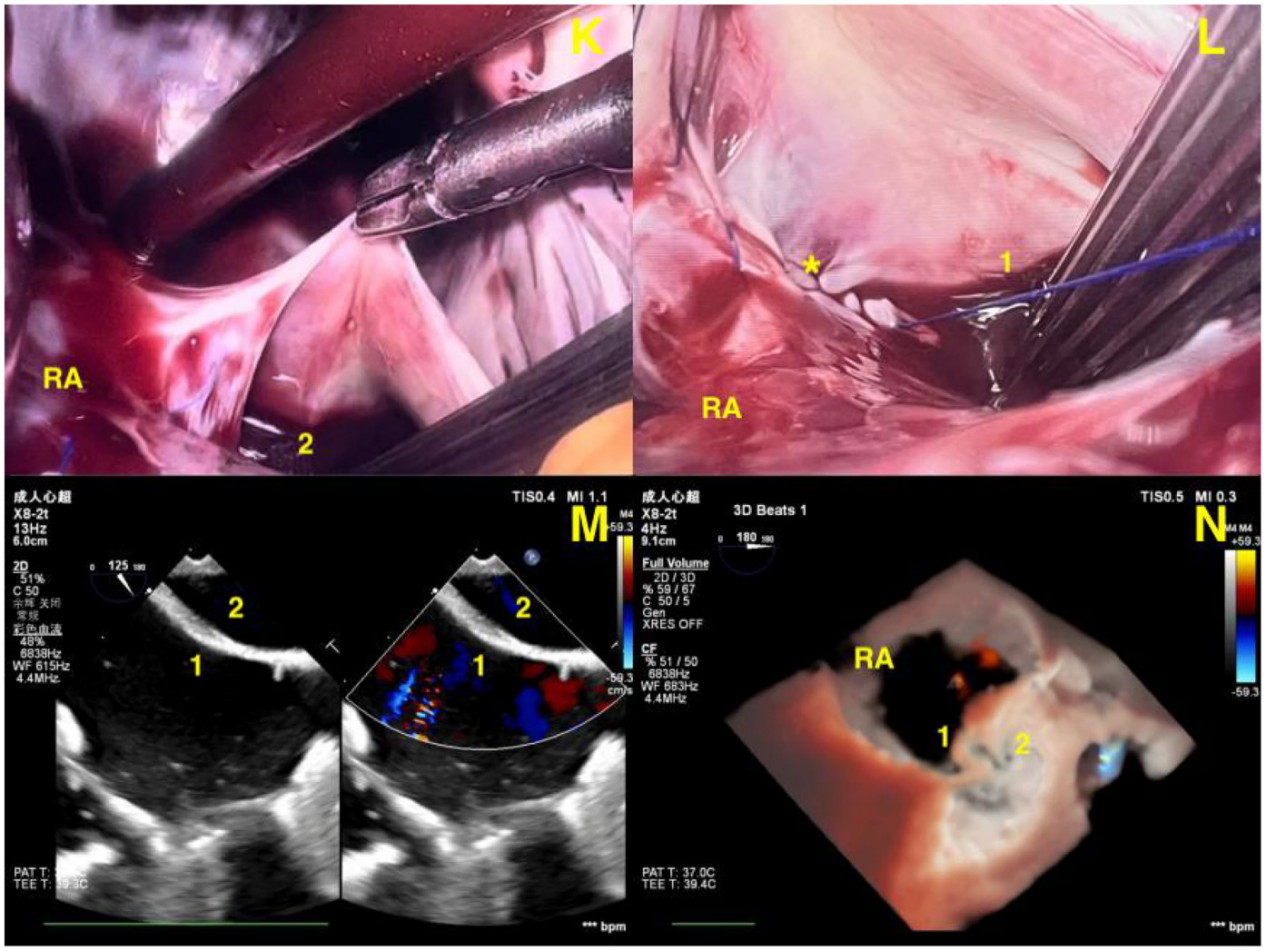
**(K,L)** Endoscopic views showing closure of the abnormal channel (*) using continuous sutures. **(M,N)** Postoperative 2D and RT-3D TEE images confirming complete closure of the RLPV–IVC communication and resolution of the abnormal shunt. (1) Inferior vena cava. (2) Right lower pulmonary vein. RA, right atrium.

After successful weaning from cardiopulmonary bypass, RT-3D TEE confirmed complete closure of the venous communication, unobstructed flow from the RLPV, and only trivial tricuspid regurgitation. No pericardial effusion or intracardiac thrombus was observed (Figures 4K–N; Supplementary Videos S5–S7). After neutralization of heparin with protamine, all cannulas were removed. Hemostasis was carefully achieved with adjunctive use of fibrin sealant. A right chest drain was placed, and the thoracic cavity was closed in layers.

The patient was extubated uneventfully and transferred to the intensive care unit in stable condition. She had an uneventful recovery, with no arrhythmias or hemodynamic instability. Follow-up echocardiography on postoperative day 3 demonstrated sustained closure of the abnormal communication, preserved pulmonary venous return, and minimal tricuspid regurgitation.

## Discussion

Sinus venosus atrial septal defect (SVASD) represents an uncommon subtype of atrial septal defects (ASDs), comprising approximately 5%–10% of cases (1). It is characterized by a defect located outside the true atrial septum, resulting from a deficiency at the junction between the systemic venous inflow—typically the superior or inferior vena cava—and adjacent pulmonary venous structures (2, 3). When the defect involves the inferior vena cava and right lower pulmonary vein (RLPV), it is classified as an inferior sinus venosus defect (ISVD), which is exceedingly rare following ASD repair (4–7). These residual anomalous venous communications may remain undetected for decades, particularly in asymptomatic individuals.

The diagnosis of ISVD is often delayed or missed, especially in adults, due to its subtle anatomical presentation and limitations of conventional imaging. While transesophageal echocardiography (TEE) is generally considered superior to transthoracic echocardiography (TTE) in identifying venous anomalies (8), the anomalous connection between the right lower pulmonary vein (RLPV) and inferior vena cava (IVC) in our patient was initially suspected on TTE and subsequently confirmed by contrast-enhanced cardiac CT. The lesion closely resembled a partial anomalous pulmonary venous return (PAPVR) or an inferior SVASD, and might have been misinterpreted as Scimitar syndrome due to overlapping imaging features.

In this case, the patient remained asymptomatic into adulthood, despite the presence of a significant residual venous communication. This may be partially attributed to her young age and preserved cardiopulmonary reserve, which allowed temporary compensation for left-to-right shunting. However, prolonged volume overload to the right atrium and right ventricle could eventually lead to progressive chamber dilation, right ventricular remodeling, and worsening pulmonary hypertension (9, 10). Timely surgical correction is therefore warranted—even in asymptomatic individuals—to prevent long-term hemodynamic deterioration and irreversible structural changes.The residual defect in this patient was likely due to incomplete suturing or dehiscence along the posterior margin during the initial repair. Reoperation was guided by echocardiographic evidence of significant shunting and right heart enlargement; cardiac catheterization was not performed. Despite initial appearances, axial contrast-enhanced CT (Figure 2F) seemed to show continuity between the inferior vena cava (IVC) and the left atrium; however, further review confirmed that this was an artifact caused by the large caliber of the IVC and slice overlap. In fact, the IVC drained normally into the right atrium without direct communication with the left atrium. This interpretation was supported by the patient's stable hemodynamics and absence of cyanosis, which would not be consistent with an IVC-left atrium drainage anomaly. Although transcatheter closure might be considered, the anatomical proximity of the residual channel to the inferior vena cava and right inferior pulmonary vein posed a risk of luminal compromise with a device, especially given the defect size. Surgical repair was therefore preferred.

Minimally invasive thoracoscopic cardiac surgery provides the benefit of reduced trauma and faster recovery. However, visualization may be impaired under certain intraoperative conditions, such as blood pooling after weaning from cardiopulmonary bypass, atypical anatomical location of the defect, or interference from electrocautery smoke. Although direct endoscopic exposure was generally sufficient, these factors intermittently limited visual clarity and increased procedural uncertainty.

In this context, real-time three-dimensional TEE (RT-3D TEE) served as a valuable intraoperative adjunct by offering dynamic, multiplanar views of cardiac anatomy. It facilitated confirmation of the RLPV–IVC communication prior to atriotomy and provided additional anatomical orientation when direct visualization was impaired. RT-3D TEE also enabled assessment of valve morphology and function, verification of guidewire positioning, and immediate post-repair evaluation of residual shunting or obstruction. These contributions improved surgical confidence and workflow efficiency.

While not essential in all thoracoscopic cardiac procedures, we believed that RT-3D TEE may offer substantial support in anatomically complex cases where spatial understanding is critical. In this case, its use supplemented—but did not replace—the thoracoscopic approach, enhancing safety and precision without disrupting the surgical process.

## Data Availability

The original contributions presented in the study are included in the article/Supplementary Material, further inquiries can be directed to the corresponding author.
